# Antiretroviral therapy non-adherence and its relationship with cognitive impairment, alcohol use disorder, and depression in adolescents living with HIV

**DOI:** 10.1186/s12888-023-05000-7

**Published:** 2023-07-24

**Authors:** Anthony A. Olashore, Bonginkosi Chiliza, Saeeda Paruk

**Affiliations:** 1 Department of Psychiatry, Faculty of Medicine, University of Botswana, Gaborone, Botswana; 2 Department of Psychiatry, Nelson R Mandela School of Medicine, University of KwaZulu-Natal, Durban, South Africa

**Keywords:** HIV, Non-adherence, Depression, Cognitive impairment, Vertical infection

## Abstract

**Objective:**

We aimed to compare antiretroviral non-adherence in the behaviourally infected (BIAs) and congenitally infected adolescents (CIAs) and explore its associations with depression, cognitive impairment, and alcohol use disorder (AUD) in adolescents living with HIV(ALWHIV) in Botswana.

**Methods:**

This study was a cross-sectional, comparative, multi-center research that involved collecting samples from different HIV clinics in Botswana. Of the 622 ALWHIV, 223 were identified as BIAs and 399 as CIAs. They were evaluated using various tools such as MINI-KID for psychiatric disorders, DSM-5 for AUD, CAT-rapid for cognitive assessment, and Visual Analogue Scale (VAS) for non-adherence (the outcome). The data were analysed using both bivariate and multivariate regression analyses.

**Results:**

The participants’ mean age (SD) was 17.7(1.60). The CIAs were more likely to have cognitive impairment (t -7.25; p < 0.01), while the BIAs had more depression (χ2 = 5.86; p = 0.016) and AUD (χ2 = 4.39; p = 0.036) and were more likely to be non-adherent (t = 3.14; p = 0.002). In the CIA group, cognitive impairment (AOR = 2.86; 95% CI:1.77–4.64) (AOR = 2.79; 95%CI:1.73–4.48) and depression (AOR = 2.69; 95%CI:1.48–4.90 were associated with ART non-adherence. In the BIA group, depression (AOR = 2.55; 95%CI:1.27–5.16), AUD (AOR = 2.58; 95%CI:1.21–5.49) and struggling to accept status (AOR = 2.54; 95%CI:1.41–4.56) predicted non-adherence to treatment.

**Conclusion:**

The two groups of adolescents differ regarding ART non-adherence and associated psychosocial issues, indicating the need for differentiated care to address non-adherence in the ALWHIV, especially in high-burden, resource-constrained settings, such as Botswana.

**Supplementary Information:**

The online version contains supplementary material available at 10.1186/s12888-023-05000-7.

## Background

The HIV infection epidemic remains a major public health challenge, with an estimated 38 million people living with the virus worldwide, affecting youth in their most productive period [1]. Eastern and Southern Africa account for more than half (54%) and two-thirds of HIV-infected children (67%), respectively [1], and despite the successful efforts to reduce the HIV infection rate since the mid-1990s, approximately 1.7 million new cases were recorded in 2019 [1]. Adolescents are the most vulnerable population, accounting for over one-third of all new HIV infections globally [1]. For example, in Botswana, a country among the top four affected by HIV, adolescents accounted for one-third of the new infection in 2018, which could be related to various psychosocial factors, including mental illness, stigma, and poor treatment adherence [2].

Antiretroviral therapy (ART) non-adherence or suboptimal use has been linked to a high rate of drug resistance, the development of more virulent strains, an increase in the rate of progression to AIDS and an upsurge of new infection [3]. Non-adherence to ART in adolescents remains a concern, with 36% reporting non-adherence in the past week in a South African study [4] and 24% in Botswana report in 2013 [5], the current rate being unknown, although it appears to be rising [2].

The elements that promote non-adherence in adolescents living with HIV (ALWHIV) are multifactorial [5], and range from the challenges associated with adolescence to other non-related adverse bio-psycho-social issues. Among these are the type of ART regimen, adverse effects, and adverse psychosocial factors, such as poverty and malnutrition [2, 5, 6]. In addition, neuro-cognitive problems, psychiatric disorders, and substance-related problems have been associated with non-adherence in ALWHIV [2, 5, 6].

The individual roles of psychiatric disorders, such as depression, substance use disorders, and cognitive disorders, on non-adherence, have been poorly identified, while their cumulative effect remains largely unexplored in ALWHIV, particularly in sub-Saharan Africa [7]. The complex interactive effect of these factors on ART non-adherence in adolescents presents an additional challenge to managing HIV/AIDS and deserves more attention [7], and calls for an integrated management model if the war against new HIV infection is to be won.

Another factor currently influencing ART non-adherence is the role of the mode of infection (MOI), with studies on neuro-cognitive sequelae of HIV suggesting the possibility of the influence of duration of exposure on cognitive impairment [8]. Hence the possibility of congenitally infected adolescents (CIAs) having more impairment due to the longer duration of exposure, regardless of treatment [8]. However, behaviourally infected adolescents (BIAs) may also have some impairment, as brain growth continues until the early 20s, but to a lesser degree [9], possibly due to a shorter duration of exposure. Conversely, the BIAs may have more psychosocial issues, such as depression and substance use, which could impact adherence and quality of life [10].

These emerging issues suggest the need for a differentiated care approach to deliver tailored, appropriate services, specifically in a low-resource country such as Botswana. Thus, we hypothesized that the two groups would differ regarding ART non-adherence, their associated factors, and psychosocial needs. This study, therefore, aimed to explore the relationship between psychiatric disorders and non-adherence and how the MOI modifies this relationship in ALWHIV in Botswana. We contend this would generate the data needed to formulate strategies to address the infected adolescents’ needs and reduce the rate of new infections in Botswana.

## Methods

### Study design and site

This cross-sectional, comparative, multi-center study was conducted among infected adolescents aged 12 to 19 years at public sector HIV clinics in Botswana. An earlier manuscript has reported details regarding the study site, sampling, and sample population [11].

### Study population and sampling

The study included only ALWHIV who had been attending the clinic for six months, could communicate in English or Setswana, and were not intellectually disabled or physically ill to consent and participate. The sample size was estimated using the formula for comparing proportions in two groups [12], with a minimum of 245 per group being intended for the study. We adopted a convenience sampling method due to the reduced client turnout, particularly during the COVID pandemic.

### Study procedure

Data were collected by trained research assistants from clinic patients who met the eligibility criteria and were introduced to the project on clinic days after their consultation with the attending physicians. However, to avoid the influence of the therapeutic relationship on the participants’ response, none of the staff attending to them at the clinic was involved in the study thereafter. The project commenced in December 2019 and ended in December 2021 due to the disruptions caused by the COVID-19 restrictions and reduced clinic patient attendance.

### Measures

Data was collected through five questionnaires; some being self-administered, and others being completed by a trained interviewer.

**The Socio-demographic** subsection contained questions on issues such as participants’ age, religion, ethnicity, educational level and gender, and parent’s marital status. It included questions on the type and level of social support they had access to; clinical variables, such as mode of infection; age at first diagnosis (HIV), and current viral load, with other details having been reported previously [11, 13].

**The Mini International Neuropsychiatric Interview for Children and Adolescents (MINI-KID)** [14] is a brief structured clinical diagnostic interview that assesses mental and behavioral disorders according to ICD-10 and DSM-IV. This interviewer-administered instrument was only used to assess current depression in the participants and had been used previously in this population [15, 16].

**The Diagnostic and Statistical Manual of Mental Disorders (DSM-5**) [17] was used to assess alcohol use disorders (AUD). Those who met at least two of the criteria listed by the diagnostic tool were reported as having AUD, regardless of the specifiers [18].

**The Cognitive Assessment Tool-rapid version (CAT-rapid)** [19] was administered and consists of four questions: on cognitive symptoms, registration of four words, a mini-trail-making test of four letter/number pairs, and word recall. CAT-rapid displayed fair sensitivity (78%) in screening for symptomatic impairment, with a score of < 10 indicating cognitive impairment [19].

**The Visual Analogue Scale (VAS)** was used to measure non-adherence, the outcome variable, and has been described in an earlier manuscript [20]. In this study, the VAS is depicted as a 10 cm long horizontal line, marked in 5 cm increments from 0 to 100. The participants had to mark their adherence levels for the past 30 days on a line chart, which included taking the medication at the right time and dose as prescribed [21]; any score below 95% was considered as suboptimal adherence or non-adherence [22]. This tool has been extensively utilized in low- and middle-income nations and has demonstrated significant (r = 0.5–0.7) correlations with other self-reported assessments, objective pill counts, electronic drug monitors (EDMs), and viral loads. (r = 0.35) [23].

### Data analysis

The data collected was entered in Statistical Package for Social Sciences (SPSS for Windows 21), Version 21. For the descriptive statistics, the continuous socio-demographics (e.g., age), the VAS, and CAT-rapid scores were presented with means and standard deviations, while the categorical variables (e.g., gender) were reported as percentages. The chi-square tests were used to present the differences between the CIA and BIA groups for categorical variables, while the independent t-tests were used to present the mean difference for the continuous variables, such as age and cognitive screening score. The VAS score was categorized into optimal and suboptimal/non-adherence for further analysis, as it was not normally distributed. We used a logistic regression model to explore the relationship between mental disorders (depression, alcohol use disorder, cognitive impairment) and non-adherence while controlling for all the socio-demographic and clinical variables that were shown to be associated with the outcome in the literature [6]. First, a bivariate model was used to assess the relationship between the independent variables and the outcome. All the variables with a p-value of ≤ 0.2 on bivariate analysis were then entered together into a multivariate regression model to explore their relationships with the outcome, this being done and presented differently for the CIAs and BIAs. A single-level multivariate logistic regression was performed while controlling for all the covariates. The Hosmer-Lemeshow goodness of fit test for logistic regression was performed, with less than 0.05 accepted as a good fit, and the level of statistical significance for all tests was set at p < 0.05.

### Ethical considerations

The protocol was approved by the University of KwaZulu-Natal Biological Research Ethics Committee and the University of Botswana’s institutional review board (UBIRB).

## Results

### Socio-demographic characteristics

For the 622 completed responses that were analyzed, the mean age (SD) was 17.7 (1.60) years. More (338, 54.3%) males than females (284, 45.7%) participated, with most being Christian (80%) belonging to the Mokgatla ethnic group (103, 16.6%). The CIAs and the BIAs were alike in their socio-demographic characteristics, except that the BIA participants were older than the CIA’s (t = -7.31; p < 0.01), as summarized in Table 1.

**Table 1:** The demographic characteristics of the HIV infected adolescents: CIAs vs. BIAs

Characteristics	Total sample N = 622 N (%)	CIA N = 399 N (%)	BIA N = 223 N (%)	p-value
**Mean age in years (SD)** ^**#**^	17.7(1.60)	17.4(1.80)	18.2(1.01)	**< 0.01**
Gender*				
Male	338 (54.3)	218 (54.6)	120 (53.8)	0.843
Female	284 (45.7)	181 (45.4)	103 (46.2)	
Religion*				
Christianity	496(80.0)	318(79.9)	178(80.2)	0.907
Other	31(5.0)	19(4.8)	12(5.4)	
No religion	93(15.0)	61(15.3)	32(14.4)	
Highest level of education*				
Junior high school and below	372(60.8)	239 (60.7)	133(61.0)	0.932
Senior High school and above	240(39.2)	155(39.3)	85(39.0)	
Perceived social support from family*				
Poor	344(55.7)	224(56.6)	120(54.1)	0.547
Good	274(44.3)	172(43.2)	102(45.9)	
Types of caregivers*				
Single parent and others	402(64.6)	253(63.4)	149(66.8)	0.394
Both parents	220(35.4)	146(36.6)	74(33.2)	

Significant p values are in bold, CIAs: Congenitally Infected Adolescents, BIAs: Behaviourally Infected Adolescents, ^#^T-test, *Chi-square

The CIAs were significantly less likely to know their HIV status (FET; p < 0.01), more likely to have other family members with HIV infection (χ2 = 36.5; p < 0.01), and to have cognitive impairment (t -7.25; p = < 0.01). On the other hand, the BIAs were less likely to accept their HIV status (χ2 = 41.2; p < 0.01) and more likely to complain when they felt worse or experienced side effects, although the difference was not significant (χ2 = 0.41; p = 0.521). The BIAs were also more likely to have depression (χ2 = 5.86; p = 0.016) and AUD (χ2 = 4.39; p = 0.036) (Table 2).

### The pattern of adherence in the ALWHIV

The mean viral load (SD) was 2104.3 (13306.4) copies/ml, and the BIAs were more likely to have a viral failure (t = -1.98; p = 0.048). The viral load score correlated well with the adherence score (ρ = -0.31; p < 0.01), with the mean adherence score (SD) being 86.7 (18.1) and almost half (44%) having been non-adherent in the past 30 days. However, the BIAs were more likely to be non-adherent to ART, with a mean score (SD) of 85.0 (17.2) (t = 3.13; p = 0.002) (Table 2).

**Table 2:** The Clinical variables of the HIV infected adolescents: CIAs vs. BIAs

Characteristics	Total sample N = 622 N (%)	CIA N = 399 N (%)	BIA N = 223 N (%)	p-value
Support/counselling from care providers*				
Poor	162(26.2)	105(26.4)	57(25.8)	0.859
Good	456(73.8)	292(73.6)	164(74.2)	
Clinic attendance*				
Poor	60(9.7)	33(8.3)	27(12.2)	0.116
Good	558(90.3)	364(91.7)	194(87.8)	
Acceptance of status*				
Difficulty in accepting status	194(33.8)	85(23.9)	109(50.0)	**< 0.01**
Has accepted status	380(66.2)	271(76.1)	109(50.0)	
**Knows HIV status** ^**@**^				
yes	545(88.0)	324(81.4)	221(100)	**< 0.01**
No	74(12.0)	74(18.6)	-	
Family member with HIV*				
Yes	406(66.8)	288(74.4)	118(53.4)	**< 0.01**
No	110(18.1)	44(11.4)	66(29.9)	
Don’t know	92(15.1)	55(14.2)	37(16.7)	
Felt worse when taking meds*				
Yes	29(4.7)	17(4.3)	12(5.4)	0.521
No	591(95.3)	381(95.7)	210(94.6)	
Depression*				
Present	147(23.6)	82(20.6)	65(29.1)	**0.016**
Absent	475(76.4)	317(79.4)	158(70.9)	
Alcohol Use Disorder*				
Present	110(17.7)	61(15.3)	49(22.0)	**0.036**
Absent	512(82.3)	338(84.7)	174(78.0)	
**Adherence score (SD)** ^**#**^	87.9(17.3)	89.6(17.1)	85.0(17.2)	**0.002**
**Viral load (SD)** ^**#**^	2104.3 (13306.4)	1302.7 (29664.4)	3552.5 (21387.0)	**0.048**
**Cognitive screening score (SD)** ^**#**^	10.17(1.67)	9.81(1.82)	10.80(1.09)	**< 0.01**

Significant p values are in bold, CIAs: Congenitally Infected Adolescents, BIAs: Behaviourally Infected Adolescents, ^#^T-test, *Chi-square, ^@^Fisher’s Exact Test

### The association of depression, cognitive impairment, and alcohol use disorder with non-adherence

Regardless of the MOI, the bivariate logistic regression analysis was used to assess the relationship between the outcome and the independent variables. Variables with a p-value of ≤ 0.2 were entered into the logistic regression model. The model revealed an association between depression (AOR = 2.51; 95%CI:1.63–3.88), impaired cognitive screening (AOR = 1.82; 95%CI:1.20–2.74), struggling to accept status (AOR = 1.52; 95%CI: 1.04–2.22) and non-adherence to ART. Also, feeling better and healthy when taking medication (AOR = 2.63; 95%CI: 1.75–3.94) was associated with non-adherence, but no relationship was found between this outcome and AUD (AOR = 1.37; 95%CI:0.86–2.18) (Supplementary Table 1).

Variables with a p-value of ≤ 0.2 on bivariate analysis were entered into the model. Tables 3 and 4 show the multivariate logistic regression model for the association of depression, impaired cognitive screening, and AUD with the outcome in the CIAs and the BIAs. After controlling for the effect of identified socio-demographic and clinical variables in the CIA group, impaired cognitive screening (AOR = 2.86; 95% CI:1.77–4.64) and depression (AOR = 2.69; 95%CI:1.48–4.90) were two times more likely to predict non-adherence. Furthermore, those who feel better and healthy when taking their medications (AOR = 3.68; 95%CI: 2.11–6.41) were also likely to be non-adherent to ART.

**Table 3:** Logistic regression model showing the predictors of adherence in the 399 CIAs.

Characteristics	N/n	COR	95% CI.		*P*	AOR	95% CI.	
			lower	Upper			Lower	Upper
Level of education*	**394**							
Junior high school and below	239	0.74	0.49	1.13	0.286	0.77	0.47	1.25
Paternal orphaned*	**389**							
Yes	76	0.48	0.27	0.84	0.701	1.38	0.27	7.12
**Maternal orphaned**	**399**							
Yes	124	0.78	0.50	1.22	0.680	0.85	0.40	1.81
**Double orphan**	**399**							
Yes	76	1.83	1.06	3.18	0.113	4.52	0.69	29.3
**Types of caregivers**	**399**							
Single parent or others	253	1.28	0.84	1.95	0.055	1.70	0.99	2.91
**Felt healthy when taking med**	**399**							
Yes	96	4.182	2.58	6.79	**< 0.01**	3.68	2.11	6.41
Felt worse when taking meds*	**398**							
Yes	17	4.09	1.41	11.9	0.224	2.09	0.64	6.81
**Cognitive screening score**	**399**							
Lower score	145	3.14	2.05	4.80	**< 0.01**	2.86	1.77	4.64
**Depression**	**399**							
Present	82	3.42	2.06	5.66	**0.001**	2.69	1.48	4.90

Significant p-values are in bold. CIAs: Congenitally Infected Adolescents, COR: crude odd-ratio, AOR: Adjusted odd-ratio, p: p-value. N/n: the overall number of participants in each category/number of the observed, * overall number of participants not equal to 399 due to missing data

**Table 4:** Logistic regression model showing the predictors of adherence in the 223 BIAs.

Characteristics	N/n	COR	95% CI.		*P*	AOR	95% CI.	
			Lower	Upper			Lower	Upper
**Gender**	**223**							
Male	120	0.57	0.33	0.97	0.093	0.59	0.32	1.09
**Maternal orphaned**	**223**							
Yes	48	1.50	0.78	2.90	0.244	1.54	0.75	3.16
Felt healthy when taking med*	**222**							
Yes	71	1.98	1.104	3.57	**0.024**	2.10	1.10	4.00
**Alcohol Use Disorder**	**223**							
Present	49	2.22	1.13	4.36	**0.014**	2.58	1.21	5.49
**Depression**	**223**							
Present	65	3.21	1.70	6.05	**0.009**	2.55	1.27	5.16
HIV status*	**218**							
Difficulty in accepting status	109	2.67	1.54	4.62	**0.002**	2.54	1.41	4.56

Significant p-values are in bold, BIAs: Behaviourally Infected Adolescents, AOR: Adjusted odd-ratio, COR: crude odd-ratio, p: p-value, N/n: the overall number of participants in each category/number of the observed, * overall number of participants not equal to 223 due to missing data

In the BIA group, depression (AOR = 2.55; 95%CI:1.27–5.16) and feeling better and healthy when taking medication (AOR = 2.10; 95% CI:1.10-4.00) remained significant predictors of non-adherence. In addition, those who had AUD (AOR = 2.58; 95%CI:1.21–5.49) and those struggling to accept their HIV status (AOR = 2.54; 95%CI:1.41–4.56) were two times more likely to be non-adherent to their medications.

## Discussion

The study explored the role of depression, cognition impairment, and AUD on non-adherence to ART in ALWHIV by MOI. As previously reported [10], the BIAs were significantly less likely to adhere to treatment and attend clinics than their CIA counterpart, possibly due to their older age, less experience, and poor adjustment to antiretroviral medication use. These findings also reflect key differences between the CIA and BIA groups regarding their expectations, needs, and support, and highlight the need for differentiated and targeted interventions amongst ALWHIV [10, 24], particularly where managing meagre available resources is an issue, such as in Botswana.

The relationship between mental disorders and non-adherence to ART is grounded in the principle of the ‘weakness of will,’ as proposed by Davidson [25, 26], and the theory of ‘health belief’ and ‘illness perception’ [27]. The theory attempted to explain why patients find it challenging to comply with prescribed therapy. Davidson described the lack of willpower as being at the core of non-adherence to ART, ‘the weakness of will,’ where an individual acts in dissonance to their best judgment [25, 26]. Mental disorders, particularly those that affect judgment, weaken this will and influence health beliefs and perceptions [26, 27]. As described by this guided theory [25, 26], we attempted to explore the relationship of three commonly identified factors influencing adherence rate among ALWHIV: alcohol use disorder, cognitive impairment, and depression [10], with the latter two being associated with non-adherence, regardless of the MOI. After controlling for the effects of the socio-demographic variables and MOI, we found that the two groups of ALWHIV had some similarities and differences regarding adherence-related factors.

Depression remained a common factor that affected adherence in both groups, as participants with depressive symptoms were two times more likely to be non-adherent than those without the symptoms. Depression has been related to ART non-adherence in various HIV-infected populations in adults [28] and adolescents [10, 29]. It is associated with poor judgment, poor concentration, and lack of motivation, leading to non-adherence to prescribed therapy [25, 26, 30–32]. Hence, healthcare providers should screen routinely for depression in adolescents suspected to be non-adherent, regardless of the MOI of HIV infection. In addition, routine screening for depression symptoms should be integrated into the care plan of ALWHIV, as it may also help prevent poor medication use.

Contrary to what was previously reported in the PLWHIV [33], those who felt healthy and had not had adverse effects since they had been on medications were more likely to be non-adherent to ART in this cohort. To the best of our knowledge, this construct is yet to be fully explored in studies regarding ART adherence among adolescents. However, a qualitative study in HIV-infected adults reported that feeling better was a barrier to non-adherence in patients taking ART in South Africa [34]. It is common practice to take ‘no present complaint’ as a sign of good response to treatment and quickly dismiss clients, especially where there is limited personnel who need to see many clients in a busy clinic. Early signs of relapse and non-adherence are easy to miss until clients develop a full-blown illness, which is more challenging to address. While it is necessary to address the unwanted medication effects and ensure or encourage adherence among those who frequently complain of medication’s adverse effects, those who feel healthy and do not complain of side effects should also be screened for non-adherence or early signs of disease. It may be that those who do not complain about medication effects may be skipping their medications or adjusting them as they feel. However, ALWHIV should be counseled that the absence of symptoms is only a sign that they are winning the battle against HIV and not an indication that the war is over.

In the CIAs, forgetfulness was a significant factor in non-adherence to ART, as previously reported [35], with the CIAs being more affected than the BIAs, due to their longer duration of exposure to the neurotoxicity effects. While the rate of severe cognitive impairment is rare due to early exposure to ART, Mild Neurocognitive Disorder (MND) and Asymptomatic Neurocognitive impairment (ANI) continue to be observed among the CIAs [35, 36]. The relationship between non-adherence and cognitive impairment is a vicious cycle [36] in that, while cognitive impairment may make ‘following a simple regimen’ difficult for adolescents, poor medication use could worsen cognitive impairment through downward disease progression. Therefore, active screening for evidence of ANI and MND in the absence of overt dementia symptomatology in the CIAs may be necessary for early identification and the prevention of cognitive-related non-adherence. In addition, workable reminder methods should be part of the management plans for the CIAs identified with some levels of forgetfulness. Research should also focus on neuro-targeted ART regimens and preventing natural disease progression to HAND.

In the BIAs, difficulty in accepting status and alcohol use disorder were related to non-adherence. The present study found a significant proportion of the BIAs still struggling to come to terms with their HIV status compared to the CIAs, possibly because they had not lived with the infection for as long. Consequently, it is reasonable to find a significant relationship between non-adherence and status acceptance only among the BIAs. While studies are yet to explore this in the adolescent populations, a study conducted among HIV-infected adults in Botswana and outside Africa reported a negative attitude towards ART in adult patients who lived in denial of their status [37, 38]. Denial may promote a lack of drive or motivation to comply with prescribed regimens and may be related to an unfavorable course of diseases, such as HIV. In addition, this maladaptive way of dealing with the knowledge of status interferes with medication non-adherence, delays help-seeking behavior, and promotes reckless lifestyles, such as unprotected multiple sexual relations, drug use, and, ultimately, the increased spread of infections. Therefore, ALWHIV, especially the BIAs, should be periodically checked for subtle signs of denial, and supportive therapy be inculcated into their care plan.

The role of alcohol in worsening disease progression has been described previously [39]. Alcohol use increases the rate of missed appointments and forgetfulness, reduces judgment, and promotes non-adherence behavior [39, 40]. In accord with our theoretical framework [26], alcohol weakens willpower and influences illness perception, as does depression and precludes the appreciation of future rewards [32]. The theory could also be related to ‘neglect of other goals and interest’ for a momentary feeling of intoxication, listed in the DSM-5 [17]. ALWHIV intentionally skips medication even without cognitive impairment or forgetfulness at the cost of tomorrow’s healthy feeling. Hence, AUD should be suspected and investigated when there is a poor response to medication or a suspected poor attitude to treatment in the BIAs. In addition, the peculiar situation of delayed adjustment in the BIAs should inform the need to consider routine counseling for alcohol use and its effect on their treatment.

### Limitations

While the present study lacks the power to decipher the direction of causality, it supports the role of depression, AUD, and cognitive impairment in the theory of ‘weakness of will,’ poor ‘health belief,’ ‘illness perception’ and their links with ART non-adherence [25, 26]. Additionally, it suggests the need to explore these relationships with a more powered methodological design, as they may be important in addressing the contribution of non-adherence to the increasing rate of new HIV infections. Our study may also be subject to a recall bias, given that some participants may have cognitive problems. Nonetheless, we assisted the participants with their records and the color codes of their medications. Therefore, a cautious interpretation of our findings regarding cognitive assessment is advised due to the use of a screening tool, CAT-rapid, which has a low diagnostic psychometric property. Lastly, the COVID-19 pandemic may have contributed to non-adherence rates and factors, although some participants were interviewed before the national lockdown, and we ensured that the period of medication recall did not include those either during or immediately afterward.

## Conclusions

Depression and cognitive impairment were associated with non-adherence after controlling for the effect of identified socio-demographic variables in our cohort. As hypothesized, the two groups differed regarding non-adherence and the associated factors, although they shared some common sentiments, such as the influence of depression and feeling better or healthy with non-adherence. The BIAs were more likely to be non-adherent to medication than the CIAs; the factors associated with non-adherence in the former included having difficulty accepting or adjusting to their status and AUD, while low cognitive scores were related to the same outcome in the CIAs.

We recommend further research on the effect of these psychosocial factors on adherence among ALWHIV and how they can be used to formulate preventive measures against ART non-adherence. Nonetheless, our study supports the need for an integrated and tailored or needs-specific approach to healthcare services delivery in addressing non-adherence among ALWHIV, especially in high-burden but resource-constrained settings, such as Botswana.

## Electronic supplementary material

Below is the link to the electronic supplementary material.

Supplementary Material 1: Logistic regression model showing the predictors of non-adherence in 622 ALWHIV

## Data Availability

The datasets used and analyzed during the current study are available from the corresponding author upon reasonable request.
