# Supplementary material for: Antiretroviral therapy non-adherence and its relationship with cognitive impairment, alcohol use disorder, and depression in adolescents living with HIV
Source: BMC Psychiatry. 2023 Jul 24;23:532. doi: 10.1186/s12888-023-05000-7 (PMC10367307; doi:10.1186/s12888-023-05000-7)
Supplement: Supplementary file 1 — Supplementary Material 1: Logistic regression model showing the predictors of non-adherence in 622 ALWHIV [file 12888_2023_5000_MOESM1_ESM.docx]

**Supplementary table 1: Logistic regression model showing the predictors of non-adherence in 622 ALWHIV.**

| **Characteristics** | **N** | **COR** | **95% CI** | | ***p*** | **AOR** | **95% CI.** | |
| --- | --- | --- | --- | --- | --- | --- | --- | --- |
|  |  |  | Lower | Upper |  |  | **Lower** | **Upper** |
| **Age** | **622** |  |  |  |  |  |  |  |
| Older age | 276 | 1.08 | 0.98 | 1.19 | 0.439 | 1.06 | 0.92 | 1.21 |
|  |  |  |  |  |  |  |  |  |
| **Highest level of education*** | **612** |  |  |  |  |  |  |  |
| Junior high school and below | 372 | 0.76 | 0.55 | 1.06 | 0.552 | 0.89 | 0.61 | 1.31 |
|  |  |  |  |  |  |  |  |  |
| **Types of caregivers** | **622** |  |  |  |  |  |  |  |
| Single parent or others | 402 | 1.28 | 0.92 | 1.79 | 0.133 | 1.34 | 0.92 | 1.96 |
|  |  |  |  |  |  |  |  |  |
| **Felt healthy when taking med*** | **621** |  |  |  |  |  |  |  |
| Yes | 167 | 3.23 | 2.23 | 4.68 | **<0.01** | 2.63 | 1.75 | 3.94 |
|  |  |  |  |  |  |  |  |  |
| **Felt worse when taking meds*** | **620** |  |  |  |  |  |  |  |
| Yes | 29 | 2.93 | 1.31 | 6.54 | 0.345 | 1.56 | 0.62 | 3.95 |
|  |  |  |  |  |  |  |  |  |
| **Cognitive screening score** | **622** |  |  |  |  |  |  |  |
| Lower score | 165 | 1.87 | 1.30 | 2.68 | **0.004** | 1.82 | 1.20 | 2.74 |
|  |  |  |  |  |  |  |  |  |
| **Alcohol Use Disorder** | **622** |  |  |  |  |  |  |  |
| Present | 110 | 1.57 | 1.04 | 2.38 | 0.183 | 1.37 | .86 | 2.18 |
|  |  |  |  |  |  |  |  |  |
| **Depression** | **622** |  |  |  |  |  |  |  |
| Present | 147 | 2.58 | 1.69 | 3.94 | **<0.01** | 2.51 | 1.63 | 3.88 |
|  |  |  |  |  |  |  |  |  |
| **HIV status*** | **574** |  |  |  |  |  |  |  |
| Difficulty in accepting status | 194 | 1.93 | 1.35 | 2.73 | **0.032** | 1.52 | 1.04 | 2.22 |

**Significant p-values are in bold, COR: crude odd-ratio, AOR: Adjusted odd-ratio, p: p-value,** **N/n: the overall number of participants in each category/number of the observed, *Overall number of participants not equal to 622 due to missing data.**
